# InstructSee: Instruction-Aware and Feedback-Driven Multimodal Retrieval with Dynamic Query Generation

**DOI:** 10.3390/s25165195

**Published:** 2025-08-21

**Authors:** Guihe Gu, Yuan Xue, Zhengqian Wu, Lin Song, Chao Liang

**Affiliations:** 1 National Engineering Research Center for Multimedia Software (NERCMS), Wuhan 430072, China; cranegu@whu.edu.cn (G.G.); 2022302111222@whu.edu.cn (Y.X.); 2023202110068@whu.edu.cn (Z.W.); cliang@whu.edu.cn (C.L.); 2 Hubei Key Laboratory of Multimedia and Network Communication Engineering, Wuhan 430072, China; 3 School of Computer Science, Wuhan University, Wuhan 430072, China

**Keywords:** cross-modal retrieval, multimodal representation learning, large language models (LLMs), dynamic query refinement, semantic reasoning

## Abstract

In recent years, cross-modal retrieval has garnered significant attention due to its potential to bridge heterogeneous data modalities, particularly in aligning visual content with natural language. Despite notable progress, existing methods often struggle to accurately capture user intent when queries are expressed through complex or evolving instructions. To address this challenge, we propose a novel cross-modal representation learning framework that incorporates an instruction-aware dynamic query generation mechanism, augmented by the semantic reasoning capabilities of large language models (LLMs). The framework dynamically constructs and iteratively refines query representations conditioned on natural language instructions and guided by user feedback, thereby enabling the system to effectively infer and adapt to implicit retrieval intent. Extensive experiments on standard multimodal retrieval benchmarks demonstrate that our method significantly improves retrieval accuracy and adaptability, outperforming fixed-query baselines and showing enhanced cross-modal alignment and generalization across diverse retrieval tasks.

## 1. Introduction

Multimodal retrieval, which aims to accurately retrieve semantically relevant information across heterogeneous modalities such as vision and natural language, has become increasingly critical with the rapid proliferation of multimodal data on the internet. The effectiveness of cross-modal retrieval largely depends on the degree of semantic alignment and interaction between visual and textual features. Recent advances, particularly in transformer-based architectures such as BLIP2 [1], have significantly enhanced multimodal representation learning by introducing sophisticated cross-modal fusion mechanisms—most notably, the Query-Former (Q-Former) module. This module employs a fixed-length query sequence to interface visual encoders with language models, thereby enabling robust translation of visual features into a language-informed semantic space.

While transformer-based frameworks such as BLIP2 have significantly advanced vision–language retrieval through the introduction of modules like the Q-Former, these designs often employ a fixed-length query structure that introduces key limitations. Specifically, the static nature of the query sequence constrains the model’s capacity to flexibly capture fine-grained semantic cues, which is particularly detrimental in scenarios where retrieval is conditioned on diverse and complex natural language instructions [2]. In practical scenarios, instructions frequently vary in both semantic richness and syntactic structure, motivating the need for a more flexible mechanism capable of dynamically modulating attention and representational granularity in response to textual input complexity. Building upon our previous work TalkSee [3], which employed LLMs to iteratively refine user queries through natural language interaction, we further examine the limitations of fixed-query structures in handling complex multimodal retrieval scenarios [4]. To address these challenges, we propose *InstructSee*, an advanced retrieval system that extends the interactive framework of TalkSee while integrating our newly developed model architecture, *BLIP2-IDQ-Former*. Specifically, *BLIP2-IDQ-Former* augments the original BLIP2 backbone with a novel architectural component, the *Instruction-aware Dynamic Q-Former (IDQ-Former)*, designed to enhance the flexibility and expressiveness of query representations in cross-modal retrieval. Unlike conventional fixed-query approaches, including those employed in our earlier design, the IDQ-Former incorporates an instruction-sensitive mechanism that dynamically estimates both the length and semantic composition of the query sequence as a function of the input instruction. This adaptive mechanism enables the model to adjust the granularity of visual feature extraction according to the complexity and specificity of user-provided textual inputs, thereby improving the system’s capacity for fine-grained semantic alignment and enhancing retrieval performance across diverse and challenging conditions.

InstructSee supports a broad range of retrieval tasks, including image-to-image retrieval, text-to-image retrieval, and visual question answering. Each task leverages the proposed dynamic query framework to effectively align visual and textual semantics, demonstrating the system’s versatility and applicability across heterogeneous retrieval scenarios. The integration of adaptive querying enables the system to process diverse instruction types while maintaining consistent retrieval precision across tasks with varying complexity.

Building upon the semantic reasoning capabilities of LLMs, the InstructSee framework integrates an LLM-driven query construction and refinement process [5]. By conditioning query generation on natural language instructions and incorporating iterative user feedback, the model can infer implicit retrieval intent and adapt its internal representations accordingly. This feedback-guided refinement process not only enhances retrieval accuracy but also significantly improves the system’s adaptability in dynamic, user-interactive retrieval environments, allowing for progressively more precise and context-aware search outcomes.

Extensive experiments conducted on standard multimodal retrieval benchmarks demonstrate that the proposed *BLIP2-IDQ-Former* consistently outperforms fixed-query baselines, yielding superior performance in both retrieval accuracy and generalization ability. Comprehensive ablation studies further validate the effectiveness of the dynamic query-length mechanism, underscoring its critical role in enabling fine-grained semantic alignment across modalities and its contribution to the overall robustness and versatility of the InstructSee system.

In summary, the principal contributions of this work are as follows:

- Instruction-aware dynamic query generation: We propose a novel mechanism that adaptively determines the number and semantic composition of Q-Former queries based on the complexity of the input instruction.
- Integration of LLM reasoning: The framework leverages the semantic reasoning capabilities of LLMs to enhance instruction interpretation and context-aware visual–textual alignment.
- Iterative refinement via user feedback: We introduce a user-in-the-loop query refinement process that enables the model to progressively adapt to implicit retrieval intentions through multiple rounds of interaction.
- Lightweight and generalizable design: Our architecture maintains compatibility with BLIP2-based retrieval pipelines, introducing minimal computational overhead while demonstrating strong generalization across diverse retrieval tasks and datasets.

## 2. Related Work

### 2.1. Multimodal Retrieval with Transformers

Transformer-based architectures have significantly advanced multimodal retrieval by enabling rich cross-modal alignment between visual and textual modalities. Approaches such as ViT-based retrieval models trained with metric learning objectives [6] and decomposed architectures like VLDeformer [7] have demonstrated impressive retrieval accuracy and computational efficiency. These methods typically operate by projecting heterogeneous modalities into a shared semantic space using fixed interaction mechanisms. However, while effective, they generally lack the flexibility to adapt to varying instruction semantics or incorporate iterative user feedback, limiting their applicability in interactive and dynamically evolving retrieval scenarios. Our work extends this line by introducing a retrieval framework that explicitly adapts query representation based on instruction complexity and user interaction.

### 2.2. Instruction-Aware and Feedback-Driven Vision–Language Models

Recent research has explored integrating user instructions into vision–language models to enhance interpretability and task-specific alignment. Models such as InstructBLIP [8] and CrossGET [2] employ prompt-based or token-guided mechanisms to modulate visual–textual representations according to input instructions, showing promising improvements in instruction following. Our previous work, TalkSee [3], advanced this paradigm by incorporating LLMs for interactive feedback, enabling systems to generate clarification questions and update queries through natural language exchange. Nonetheless, the design of TalkSee was still limited by its reliance on a fixed-length Q-Former inherited from BLIP2, which restricted its capacity to adjust to instructions of varying semantic richness. In contrast, the present work introduces a dynamic query generation mechanism that adaptively allocates attention resources, better addressing diverse and complex retrieval tasks.

### 2.3. Semantic Reasoning with Large Language Models

The integration of LLMs has substantially advanced semantic understanding and multimodal reasoning, empowering systems to parse complex instructions and engage in flexible user interactions. Notable systems such as LLaVA [9] and MiniGPT-4 [10] combine vision encoders with pretrained LLMs, enabling open-ended dialogue and visual instruction following. While these models excel at generating conversational outputs, they often prioritize end-to-end response generation over the explicit modeling of retrieval intent, and typically lack dedicated mechanisms for refining retrieval queries based on structured feedback. Our approach builds upon the semantic reasoning capabilities of LLMs but uniquely positions them within the retrieval pipeline as active mediators, responsible for disambiguating user intent through clarifying questions and synthesizing semantically refined queries that guide iterative search.

## 3. Methodology

**Figure 1:**
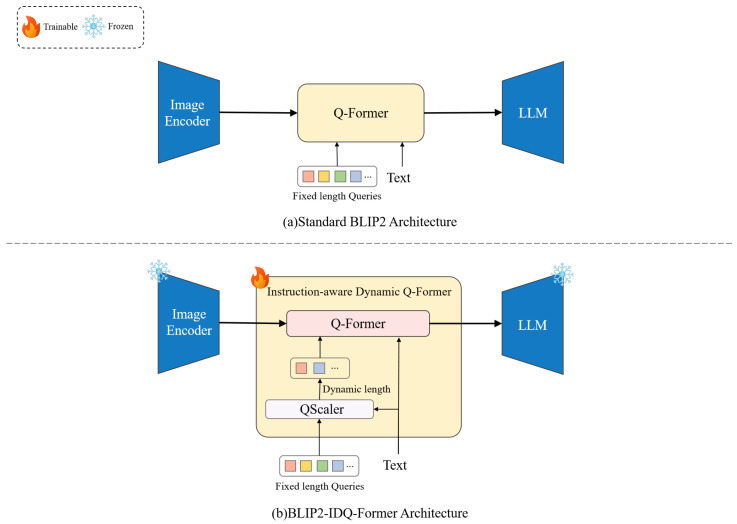
Comparison between standard BLIP2 and our proposed BLIP2-IDQ-Former. The standard BLIP2 architecture employs a static Q-Former with a fixed 32-token query sequence, regardless of instruction complexity. In contrast, the proposed *BLIP2-IDQ-Former* integrates a *QScaler* module that adaptively predicts the query length according to instruction semantics. The Q-Former processes the resulting variable-length query tokens for instruction-sensitive cross-modal encoding. During training, only the Instruction-aware Dynamic Q-Former (IDQ-Former) components are updated, while the image encoder and LLM remain frozen.

We propose *BLIP2-IDQ-Former*, an enhanced cross-modal retrieval model that extends the BLIP2 [1] framework by introducing an instruction-adaptive query mechanism. This architectural innovation serves as the core of our InstructSee system, enabling it to better accommodate diverse retrieval tasks requiring flexible and expressive visual–language alignment. Specifically, *BLIP2-IDQ-Former* integrates an *Instruction-aware Dynamic Q-Former (IDQ-Former)*, designed to dynamically allocate query capacity in proportion to the semantic complexity of user instructions, thereby improving retrieval adaptability and precision. An overview of the proposed architecture and its comparison with the standard BLIP2 design are illustrated in Figure 1.

### 3.1. Fixed-Length Query Design in BLIP2

In the original BLIP2 framework, the Q-Former is initialized with a fixed number of learnable query tokens (typically N=32) used to extract cross-modal representations from frozen image encoder outputs. This task-agnostic design applies the same query length across all inputs, regardless of instruction complexity. While stable and simple, it introduces two key limitations:

- For simple instructions, excess tokens lead to unnecessary representational redundancy.
- For complex instructions, the 32-token cap constrains the model’s capacity to capture fine-grained visual details relevant to user intent.

### 3.2. Instruction-Adaptive Token Scaling via QScaler

Conventional Q-Former architectures, such as BLIP2, allocate a fixed number of learnable query tokens regardless of the semantic complexity of the input instruction. This static allocation often leads to inefficiencies: simple instructions may be over-parameterized, wasting attention capacity, while complex instructions may be under-parameterized, limiting the model’s ability to encode fine-grained multimodal correspondences.

To address this, we propose the *Instruction-aware Dynamic Q-Former* (IDQ-Former), which augments the original BLIP2 Q-Former with a learnable instruction complexity regressor (*QScaler*) and a dynamic query token bank with positional encoding reuse. The overall architecture is shown in Figure 1, and the procedural steps are detailed in Algorithm 1. *QScaler* first estimates the complexity of the instruction and then controls the number of query tokens allocated to the Q-Former.

Given an instruction *I*, the frozen BLIP2 text encoder produces a contextual embedding $E_{\mathrm{instr}}\in R^{d}$, which is fed into *QScaler*:

$$r=\sigma \;\left(W_{2}\cdot \mathrm{ReLU}\left(W_{1}\cdot E_{\mathrm{instr}}\right)\right),\;r\in \left[0,1\right],$$

where σ(·) is the sigmoid function. During training, Gaussian noise is injected into *r* to improve robustness, followed by clipping to the range [0,1]. The predicted score *r* determines the query length as

$$N_{\mathrm{query}}=N_{\min}+\left\lfloor r\cdot (N_{\max}-N_{\min})\right\rfloor ,$$

corresponding to Step 2 of Algorithm 1.

The first $N_{\mathrm{query}}$ learnable tokens are retrieved from the token bank T, and the corresponding positional encodings are reused from the pretrained positional encoding matrix P (Step 2 of Algorithm 1). This positional encoding reuse preserves spatial and semantic alignment learned during pretraining, allowing variable-length queries without retraining positional parameters.   
**Algorithm 1:** Overall Pipeline of BLIP2-IDQ-Former**Input**: Instruction *I*, Image *V*, Base query length $N_{\min}$, Max query length $N_{\max}$**Output**: Task-specific output *O***Step 1: Instruction complexity estimation**;Tokenize *I* and encode via frozen language encoder to obtain contextual embedding $E_{\mathrm{instr}}$;Predict normalized complexity score r∈[0,1] using the learnable QScaler module;During training, apply Gaussian noise injection to *r* for robustness;**Step 2: Dynamic query allocation**;$N_{\mathrm{query}}\leftarrow N_{\min}+\left\lfloor r\cdot \left(N_{\max}-N_{\min}\right)\right\rfloor$;Select first $N_{\mathrm{query}}$ learnable tokens from token bank T;Retrieve first $N_{\mathrm{query}}$ positional encodings from pretrained matrix P;Add positional encodings to tokens to form query sequence Q;**Step 3: Cross-modal encoding**;Extract visual embeddings $E_{\mathrm{img}}$ from frozen image encoder;Fuse Q with $E_{\mathrm{img}}$ via Q-Former cross-attention;Project multimodal features into LLM input space via projection head;**Step 4: Output generation**;Feed projected features into frozen LLM;Obtain task-specific output *O*;**return** *O*;

### 3.3. Instruction-Conditioned Cross-Attention

The adaptive query sequence Q, generated according to the predicted instruction complexity, serves as the query input to the Q-Former’s cross-attention module, while the frozen image encoder outputs $E_{\mathrm{img}}$ act as the key–value pairs (Step 3 of Algorithm 1 and Figure 1). This design enables the attention mechanism to dynamically adapt its scope and focus: when Q is short, the model concentrates on a small set of high-salience visual tokens, whereas a longer Q allows the attention span to cover a broader visual context and capture more diverse semantic relations.

By conditioning the cross-attention on instruction complexity, IDQ-Former modulates both the granularity and the coverage of visual feature extraction. Shorter queries, typically triggered by simple and explicit instructions, reduce redundancy and computation while preserving task-relevant information. In contrast, longer queries, which are allocated to complex or ambiguous instructions, expand the representational capacity, enabling the capture of nuanced semantics, spatial relationships among multiple objects, and fine-grained contextual cues that are crucial for reasoning-intensive tasks.

Formally, the cross-attention output $H_{\mathrm{qf}}$ is passed through a learnable projection head $f_{\mathrm{proj}}$ to align the multimodal representation space with that of the frozen LLM:

$$H_{\mathrm{llm}}=f_{\mathrm{proj}}\left(H_{\mathrm{qf}}\right).$$

The resulting $H_{\mathrm{llm}}$ is then fed into the LLM to produce task-specific outputs, such as open-ended answers, ranked retrieval lists, or other instruction-driven predictions (Steps 3–4 of Algorithm 1). This seamless integration of QScaler-controlled token allocation and instruction-conditioned cross-attention constitutes the core of IDQ-Former’s adaptivity, providing a unified mechanism that supports efficient yet expressive multimodal reasoning across a diverse range of vision–language tasks.

### 3.4. Training Strategy

As illustrated in Figure 1, the training strategy focuses exclusively on optimizing the components within the IDQ-Former while keeping the image encoder and the LLM frozen. This design leverages the pretrained knowledge embedded in the visual and language backbones, effectively reducing the risk of overfitting and improving training efficiency.

Specifically, we updated the following modules:

- The Q-Former transformer layers responsible for cross-modal feature fusion;
- The learnable query token matrix, which provides the dynamically selected token subsets;
- The QScaler regression head, which predicts the instruction-conditioned complexity scores.

To enhance robustness and encourage smooth complexity estimation, Gaussian noise is injected into the predicted score:

$$r^{\prime }=\mathrm{clamp}\left(r+\epsilon \right),\;\epsilon ∼N\left(0,\sigma^{2}\right),$$

where the clamp function ensures the perturbed score remains within valid bounds. This regularization strategy promotes stable learning of the mapping between instruction complexity and adaptive query allocation, allowing the model to generalize more effectively across diverse tasks.

By isolating the optimization to the dynamic query mechanism and maintaining the pretrained backbone parameters, the training procedure preserves architectural modularity, enables efficient convergence, and ensures that the system remains broadly applicable to varied cross-modal retrieval scenarios.

## 4. System Overview

**Figure 2:**
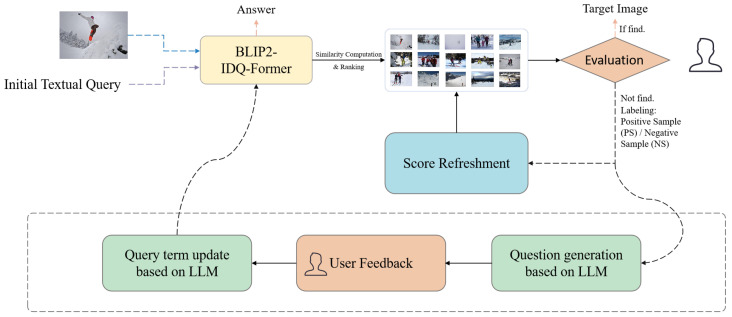
System pipeline of the proposed InstructSee. InstructSee supports text-to-image (T2I), image-to-image (I2I), and visual question answering (VQA) tasks. Task-specific input modalities are denoted with dashed arrows of different colors: purple for text input, blue for image input, and both for combined input. The framework integrates BLIP2-IDQ-Former for initial query embedding, followed by a feedback loop with score refreshing or LLM-guided refinement, which iteratively improves retrieval until the target is reached.

We present *InstructSee*, a unified instruction-aware multimodal retrieval system designed to handle complex and evolving user intent across multiple retrieval tasks. As illustrated in Figure 2, the system integrates LLMs into a human-in-the-loop framework, enabling iterative query refinement through natural language feedback [11]. Compared to traditional static retrieval pipelines, InstructSee supports dynamic adaptation via clarifying question generation, feedback-guided disambiguation, and semantic query updates, allowing flexible cross-modal alignment.

### 4.1. Task Modes and Input Interface

**Figure 3:**
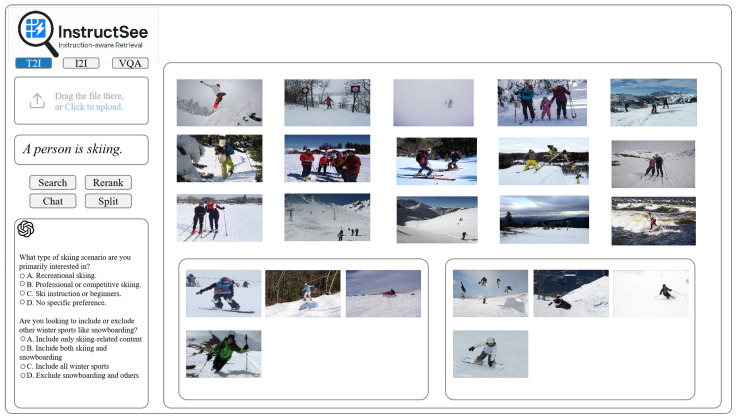
User interface of the InstructSee system. The interface supports three task modes: text-to-image, image-to-image, and visual question answering. Users can input natural language instructions or upload reference images, visualize ranked retrieval results, and interactively refine queries through feedback, reranking, and conversational clarification.

InstructSee supports three key task modes within a shared cross-modal embedding space, each addressing distinct retrieval scenarios (see Figure 3 for interface overview):

- Visual Question Answering (VQA): Given an input image and a user-issued question (e.g., “What is the person doing?”), the system directly generates an answer using its multimodal encoder-decoder, without a ranking process.
- Image-to-Image Retrieval (I2I): Provided with a reference image, the system computes cross-modal similarity scores against a gallery of pre-encoded images to retrieve visually or semantically similar samples.
- Text-to-Image Retrieval (T2I): Given a natural language description, the system encodes the textual query and ranks gallery images according to semantic similarity.

For the retrieval tasks (I2I and T2I), the system computes an initial ranked list of candidate images based on cosine similarity between the query and gallery embeddings, providing a foundation for subsequent refinement. In contrast, the VQA task directly produces an answer without requiring a retrieval stage. Task-specific input modalities are indicated using dashed arrows in Figure 2: T2I accepts only textual input, I2I uses image input, and VQA simultaneously incorporates both image and textual input.

### 4.2. User Feedback and Interactive Refinement

When the desired target image, which refers to the image that best satisfies the user’s explicit or implicit retrieval intent, is not successfully retrieved in the initial ranking, the system activates an interactive refinement loop, as illustrated in Figure 2:

Users annotate retrieved images as positive samples (PS) or negative samples (NS), signaling their relevance or irrelevance.

To incorporate user feedback, the system offers two complementary strategies:

- Score Refreshing: The system updates similarity scores by integrating the embeddings of the original query and the PS/NS samples, allowing local re-ranking without altering the semantic composition of the query.
- LLM-Guided Semantic Refinement: For more nuanced adjustment, the system employs an LLM interaction module [12], with the following features:
  - Clarifying single-choice questions are generated based on the original query and the PS/NS captions, aiming to uncover latent intent dimensions (e.g., action, object attributes, and scene context).
  - User-selected answers are incorporated to synthesize a semantically refined query.
  - The refined query $V_{{\mathrm{Qry}}_{\mathrm{new}}}$ is embedded and used for gallery re-ranking as follows:
    $${\mathrm{Score}}_{\mathrm{new}}\left(V_{i}\right)=\kappa \cdot \cos\left(V_{{\mathrm{Qry}}_{\mathrm{new}}},V_{i}\right)+\left(1-\kappa \right)\cdot {\mathrm{Score}}_{\mathrm{prev}}\left(V_{i}\right),$$
    where $V_{{\mathrm{Qry}}_{\mathrm{new}}}$ denotes the refined query embedding, $V_{i}$ is the embedding of gallery image *i*, ${\mathrm{Score}}_{\mathrm{prev}}\left(V_{i}\right)$ is the previous similarity score, and κ∈(0,1) balances the updated semantic content and the prior similarity scores.

This iterative refinement process supports progressive intent modeling and adaptive retrieval, ultimately enhancing accuracy and user satisfaction.

### 4.3. Prompt Design for Instruction Interpretation

**Figure 4:**
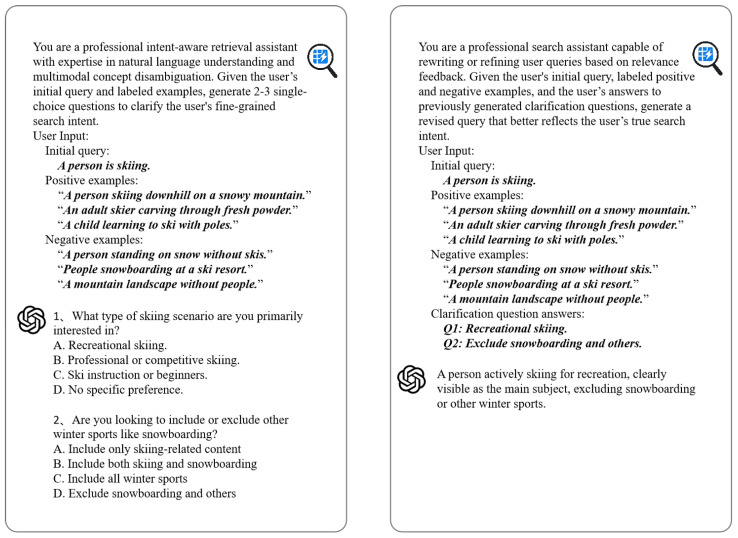
Prompt templates used for LLM-guided retrieval refinement. **Left:** The Question Generation Prompt embeds the original query and user-labeled sample captions to prompt the LLM to generate intent-disambiguating questions. **Right:** The Query Update Prompt incorporates all interaction context, original query, generated questions, answers, and captions, to produce a refined textual instruction. Template fields shown in ***bold italics*** denote dynamic placeholders to be filled with real inputs during runtime (e.g., *"A person is skiing."*).

To enable effective instruction-aware interaction, InstructSee integrates two structured prompt templates that guide the LLM in interpreting user feedback and refining retrieval intent [8,13,14]. As depicted in Figure 4, each prompt contains dynamically populated placeholders (highlighted in ***bold italics***), which are instantiated with task-specific inputs at inference time [15]. For example, the user instruction *"A person is skiing."* serves as the base query in Figure 4 to illustrate the prompt structure.

The prompt design includes two key components:

- Question Generation Prompt: This template incorporates the original query along with natural language captions of the user-labeled positive and negative samples. The LLM is instructed to generate two to three clarifying, single-choice questions that contrast salient semantic attributes (e.g., action, background, clothing), distinguishing relevant from irrelevant results [16].
- Query Update Prompt: This template extends the interaction by embedding the original instruction, the generated questions, the user-selected answers, and sample captions. The LLM uses this information to synthesize a refined, semantically enriched query that more accurately reflects the clarified retrieval objective.

These prompt strategies effectively transform implicit user feedback into explicit semantic constraints, allowing the system to refine its retrieval behavior with high precision and robust generalization across diverse task scenarios [17].

## 5. Results

### 5.1. Experimental Setup

#### 5.1.1. Datasets

We conducted experiments on three representative benchmarks spanning different application domains: COCO2014 [18], MedPix [19], and RSICAP [20]. COCO2014 is a widely used dataset for general image–text retrieval, containing over 80K training images and 40K validation images, each paired with five human-annotated captions. Following the standard Karpathy split [21], we used 113K image–caption pairs for training. MedPix is a large-scale medical image database covering diverse imaging modalities and clinical cases, with each image accompanied by descriptive textual annotations. RSICAP is a remote sensing image–caption dataset containing high-resolution aerial images paired with detailed scene-level descriptions. All models were trained on COCO2014 in a contrastive learning setting using only image–caption pairs and were evaluated on MedPix and RSICAP in a zero-shot manner to assess cross-domain generalization without external fine-tuning.

#### 5.1.2. Evaluation Strategy

We evaluated the proposed *BLIP2-IDQ-Former* on both in-domain and cross-domain settings. For the in-domain evaluation, retrieval performance was measured on the COCO2014 validation split [18]. For the cross-domain evaluation, we directly applied the COCO-trained model to MedPix [19] and RSICAP [20] in a zero-shot manner, without any task- or domain-specific fine-tuning.

In all cases, we compute the cosine similarity between the encoded instruction features and image features and report retrieval metrics accordingly. Additionally, we analyzed how retrieval performance varies with the dynamically predicted query length, enabling a direct assessment of the model’s efficiency and adaptiveness across different instruction complexities [22].

#### 5.1.3. Baselines

We compared the proposed *BLIP2-IDQ-Former* against four representative vision–language or adaptive inference baselines:

- CLIP [23]: a dual-encoder architecture trained on large-scale image–text pairs, widely adopted for zero-shot image–text retrieval;
- BLIP2 [1]: employs a fixed-length Q-Former with 32 learnable query tokens for vision–language alignment and serves as the primary backbone for our approach;
- DynamicViT [24]: adapts token usage dynamically based on visual input, reducing computational redundancy in vision transformers;
- CrossGET [2]: a cross-modal retrieval model that leverages global–local attention for enhanced vision–language matching.

Our method replaces the fixed query design in BLIP2 with an instruction-aware query scaling mechanism. A lightweight *QScaler* module predicts the optimal query length for each instruction, and the Q-Former operates on the resulting variable-length query sequence. During training, only the *QScaler* and Q-Former parameters are updated, while all other components remain frozen to ensure a fair and controlled comparison with the fixed-query and other adaptive baselines.

#### 5.1.4. Implementation Details

Our model was implemented based on the publicly available BLIP2 framework. The Q-Former module consists of 12 transformer layers with a hidden size of 768, initialized from the pretrained BLIP2 Q-Former. The instruction complexity regressor was implemented as a lightweight two-layer MLP with ReLU activation, which mapped the [CLS] token embedding from a frozen BERT encoder into a scalar score r∈[0,1] representing instruction complexity. Based on this score, we dynamically allocated a variable number of query tokens, with the total query length ranging from a minimum of 4 tokens to a maximum of 32 tokens.

Both the image encoder (ViT-L/14) and the language model (OPT-2.7B) were kept frozen throughout training. Only the Q-Former and the *QScaler* module were updated. We trained the model using the AdamW optimizer with a learning rate of $1\times 10^{-5}$. All training and inference were conducted on a single NVIDIA RTX 4090 GPU (NVIDIA, Santa Clara, CA, USA). A summary of the training configuration is provided in Table 1.

### 5.2. Quantitative Results

#### 5.2.1. Evaluation Metrics

We assessed retrieval performance in both in-domain (COCO2014 validation split [18]) and cross-domain (MedPix [19] and RSICAP [20]) settings. For each dataset, we computed the cosine similarity between the encoded instruction embedding and the corresponding ground-truth image embedding. We report the average and maximum similarity scores across all query–image pairs to capture both overall and peak retrieval quality.

To evaluate the efficiency of the proposed dynamic query allocation, we also measured the average number of query tokens predicted by the *QScaler* module over the entire test set, enabling direct analysis of the trade-off between retrieval accuracy and computational cost.

#### 5.2.2. Performance Comparison

As summarized in Table 2, the proposed *BLIP2-IDQ-Former* consistently outperforms the BLIP2 baseline in the in-domain COCO2014 benchmark, achieving an average similarity score of 0.9411 compared to 0.8569 for BLIP2. This improvement is accompanied by a competitive maximum similarity and is obtained with a dynamic average of 17.8 query tokens, substantially lower than the fixed 32 tokens in BLIP2, thereby validating the efficiency of the proposed instruction-adaptive query scaling.

In the cross-domain evaluation on MedPix and RSICAP, *BLIP2-IDQ-Former* maintains competitive retrieval accuracy, indicating a degree of robustness to domain shift. While the model exhibits slight performance variations across datasets—likely due to the distributional differences between COCO and the specialized medical and remote sensing domains—it still achieves strong overall retrieval quality without task-specific fine-tuning.

Notably, the proposed method achieves this performance with moderate memory usage relative to other high-performing baselines (e.g., CrossGET) and a throughput comparable to DynamicViT, demonstrating that adaptively controlling query length can balance accuracy and computational efficiency in both in-domain and zero-shot retrieval settings.

#### 5.2.3. Practical Feedback Protocol and Convergence Analysis

To address the concern regarding the undefined feedback mechanism, we implemented a practical interactive feedback protocol that sequentially combines two complementary interaction modes:

- Score Refreshing: The system updates similarity scores by integrating the embeddings of the original query with those of positive/negative samples (PS/NS), enabling local re-ranking while preserving the semantic composition of the query.
- LLM-Guided Semantic Refinement: The system employs an LLM interaction module to generate semantically refined query variants, which are re-encoded and re-ranked to better capture nuanced retrieval intents.

During retrieval, the protocol alternates between the two modes: Score refreshing is first applied to exploit fine-grained local re-ranking without semantic drift, and LLM-guided semantic refinement is invoked when larger semantic adjustments are required. This iterative combination continues until the target image is retrieved.

To quantify convergence behavior, we designed a benchmark of 20 query targets spanning multiple complexity levels. For each target, we recorded the cumulative number of interactions from both modes until convergence, where convergence is defined as the first occurrence of the target image within the top-5 ranked results. The results show an average of 0.90 LLM-guided refinements and 1.75 score refreshes per query to reach the retrieval goal.

This analysis highlights that the proposed dual-mode feedback protocol achieves efficient convergence by leveraging the complementary strengths of local score adjustments and semantic-level query reformulation, offering both adaptability and low interaction cost compared to static re-ranking methods such as CLIP or BLIP2 logits.

### 5.3. Ablation Study

To investigate the effectiveness of individual components in our proposed *BLIP2-IDQ-Former* framework, we conducted two ablation studies: (1) we examined the impact of the *QScaler* module for dynamic token scaling and (2) evaluated whether training the Q-Former yields additional benefits.

#### 5.3.1. Effect of QScaler-Guided Query Length Adaptation

We first evaluated the influence of the *QScaler* module by comparing the *BLIP2-IDQ-Former* with and without QScaler-enabled dynamic query length adaptation while keeping the Q-Former trainable in both settings. Table 3 summarizes the results.

*QScaler* adapts the number of query tokens to the semantic complexity of the instruction and yields a measurable improvement in cross-modal alignment. On COCO val2014, the dynamic variant attains a higher mean similarity than the fixed-length baseline. A paired bootstrap test over per-sample similarity with 10,000 resamples confirms statistical significance, with improvement Δ=+0.0039 and 95% confidence interval [0.0036,0.0043] and $p\;<\;10^{-6}$. The learned allocation concentrates queries on instruction-relevant cues and produces shorter sequences on average (mean query length 17.8), which reduces per-sample latency.

From an efficiency perspective, *QScaler* slightly reduces computation on the vision encoding plus Q-Former path and yields a small but consistent throughput gain. Using fvcore on the *encode_image* path (vision encoder and Q-Former), the dynamic setting at median query length reports 258.29 GFLOPs compared to 259.63 GFLOPs for the fixed-length baseline. Throughput measured with batch size 1 in fp16 is 21.85 versus 21.66 images per second. These modest FLOP savings are expected because the vision backbone dominates the budget, while *QScaler* primarily affects the cost of cross-attention through the number of queries. The latency reduction observed in the ablation table is aligned with this effect.

Regarding retrieval accuracy, rank-sensitive metrics are marginally lower under dynamic queries (R@5: 0.34 vs. 0.35; MRR: 0.25 vs. 0.26). This outcome is consistent with the fact that shorter queries encode a narrower range of visual evidence and may omit fine-grained cues, an effect that becomes pronounced when candidate images are highly similar and disambiguation requires more exhaustive visual encoding. In practice, this accuracy–efficiency trade-off can be mitigated by increasing the minimum query budget for difficult cases or by calibrating the complexity predictor with a recall-aware objective, thereby retaining the latency and efficiency benefits while safeguarding fine-grained retrieval performance.

Overall, these findings highlight the strength of our proposed approach in achieving semantically adaptive and computationally efficient retrieval while also pointing to the need for interactive feedback mechanisms in visually dense search environments.

#### 5.3.2. Effect of Training the Q-Former

We assessed the effect of making the Q-Former trainable by comparing two settings: updating its parameters during training versus freezing it while keeping all other components identical. Results are summarized in Table 4.

Enabling Q-Former training yields clear gains in retrieval accuracy. On COCO val2014, the trainable configuration attains a higher mean similarity than the frozen counterpart, and a paired bootstrap test over per-sample similarity with 10,000 resamples confirms statistical significance, with improvement Δ=+0.0071, 95% confidence interval [0.0068,0.0073], and $p\;<\;10^{-6}$. Rank-sensitive metrics follow the same trend, with R@5 increasing from 0.27 to 0.34 and MRR increasing from 0.20 to 0.25 under the trainable setting. These results indicate that fine-tuning enables the Q-Former to specialize its cross-modal attention and produce query embeddings that are better aligned with instruction semantics.

The efficiency profile remains essentially unchanged. The average query length is slightly larger for the trainable variant (mean 17.8 versus 16.5 for frozen; medians 18 versus 16), reflecting a modest expansion of cross-attention budget. FLOPs on the *encode_image* path (vision encoder and Q-Former, measured with fvcore) are nearly identical, with median-query estimates of 258.29 GFLOPs for trainable and 258.10 GFLOPs for frozen, a difference below 0.1%. Throughput at batch size 1 in fp16 is also comparable (21.85 versus 21.89 images per second). The per-sample latency reported in the ablation table shows a small reduction for the trainable configuration (0.06 s versus 0.07 s).

Overall, these findings support jointly optimizing the Q-Former within the dynamic query framework. Fine-tuning improves cross-modal alignment and retrieval quality while leaving computational cost and throughput effectively unchanged, implying that the efficiency budget is dominated by the vision backbone rather than the cross-attention interface.

## 6. Discussion

This work explores instruction-aware adaptation in cross-modal retrieval, focusing on the dynamic modulation of query representation conditioned on the semantic complexity of user-provided inputs. Our proposed *BLIP2-IDQ-Former* introduces a learnable query control mechanism that adjusts the number of visual queries in response to natural language instructions, providing improved semantic alignment and better utilization of model capacity. We discuss key aspects of our findings below.

### 6.1. Instruction-Aware Query Allocation Improves Semantic Alignment

Our results indicate that a fixed number of query tokens, as used in BLIP2 [1], is suboptimal for diverse retrieval scenarios. In particular, fixed-length query embeddings may be overcomplete for simple instructions and insufficient for complex ones. By conditioning the query length on instruction complexity, *BLIP2-IDQ-Former* allows the model to allocate attention capacity more appropriately across tasks. This results in improved retrieval performance across the spectrum of input complexity.

### 6.2. Joint Optimization of Q-Former and QScaler Is Essential

Our ablation analysis confirms that training the Q-Former in conjunction with the *QScaler* is critical for performance. While *QScaler* alone can predict appropriate query lengths, the Q-Former must be adapted to operate effectively under variable-length configurations. Without this joint training, the model suffers from representational mismatch and reduced alignment accuracy.

### 6.3. Token Efficiency and Inference Trade-Offs

While BLIP2-IDQ-Former substantially reduces the average number of query tokens required per instruction, this does not necessarily imply a reduction in overall inference cost. The introduction of the *QScaler* module and dynamic token allocation logic adds computational overhead compared to static pipelines such as BLIP2 [1]. Nonetheless, the observed improvement in retrieval accuracy—achieved with fewer tokens—suggests that semantically informed token allocation is a more efficient use of representational capacity. This trade-off highlights a promising direction for future work on resource-aware multimodal retrieval, particularly in balancing adaptive modeling with deployment efficiency.

### 6.4. Extensibility Beyond Retrieval

Although our model is evaluated in the context of image–text retrieval, the instruction-aware query adaptation mechanism is broadly applicable to other multimodal tasks, such as visual question answering, caption generation, or instruction-conditioned video understanding. Its modularity allows seamless integration into any encoder–decoder framework where visual grounding conditioned on language is required.

### 6.5. Ethical Considerations and Robustness

From an ethical and robustness standpoint, dynamic querying systems inherently risk amplifying biases, particularly when spurious correlations present in the training data are reinforced through LLM-mediated feedback. Such reinforcement can propagate misinformation, perpetuate stereotypes, and influence downstream decision-making.

To provide an initial robustness assessment, we constructed two categories of evaluation queries with distinct complexity profiles: (i) low-complexity queries, which are short, syntactically simple, and contain explicit retrieval targets but limited contextual detail, thereby exhibiting higher semantic ambiguity, and (ii) high-complexity queries, which involve longer multi-clause constructions, implicit or context-dependent retrieval intent, and require cross-referencing multiple concepts, thus offering richer semantic cues.

We measured the average number of combined interaction rounds required to successfully retrieve the user’s intended target. Results show that low-complexity queries required an average of 1.20 interactions, whereas high-complexity queries required only 0.80 interactions, corresponding to a 33.3% reduction in interaction effort. This outcome indicates that, despite their structural simplicity, low-complexity queries often lack sufficient discriminative information, necessitating additional rounds of clarification to resolve ambiguity. Conversely, high-complexity queries, while syntactically more elaborate, tend to encode more precise semantic constraints, enabling the system to converge more quickly on the correct retrieval target.

Building on these findings, our future work will expand robustness evaluation to encompass adversarial instruction testing with deliberately crafted prompts, as well as large-scale audits spanning multilingual, culturally diverse, and domain-specific scenarios. In parallel, we plan to integrate fairness-aware ranking mechanisms and multi-source verification of LLM-generated responses, further enhancing both the ethical safety and practical resilience of dynamic querying systems.

### 6.6. Limitations and Future Work

A limitation of the current design is its reliance on a learned scalar predictor to estimate instruction complexity, which may underperform in domain-shift scenarios or highly ambiguous queries. Moreover, while the query length is adaptively adjusted, the allocation of query content remains uniform. Future extensions may explore fine-grained attention reallocation across tokens or structured query generation conditioned on syntactic or semantic cues. Additionally, incorporating user interaction history or iterative refinement strategies may further improve retrieval robustness.

## 7. Conclusions

In this work, we present *BLIP2-IDQ-Former*, an instruction-aware cross-modal retrieval framework designed to dynamically adapt query representations in accordance with the semantic complexity of natural language instructions. Built upon the BLIP2 architecture [1], the proposed framework integrates a learnable *QScaler* module for predicting instruction-conditioned query lengths and a fine-tuned Q-Former for processing variable-length query embeddings. This adaptive design enables the model to generate retrieval representations that are both efficient and semantically expressive, allowing for more precise alignment with diverse and user-specific retrieval intents.

Through extensive experiments on COCO2014 [18], we demonstrate that *BLIP2-IDQ-Former* significantly improves retrieval accuracy compared to fixed-query baselines, while using substantially fewer query tokens on average. Our results validate the importance of instruction-conditioned token allocation and highlight the limitations of static visual querying strategies in handling diverse semantic input.

The proposed framework is lightweight, modular, and compatible with existing multimodal pipelines, making it broadly applicable to other instruction-guided vision–language tasks. Future work will explore extending the instruction-aware query paradigm to hierarchical or structured token representations and enhancing generalization in open-domain or user-interactive retrieval settings.

## Figures and Tables

**Table 1:** Training and evaluation configuration of our proposed BLIP2-IDQ-Former model.

Configuration	Value
Vision Encoder Initialization	Pretrained BLIP2 ViT-L/14
LLM Initialization	Pretrained OPT-2.7B (frozen)
Q-Former Initialization	BLIP2 Q-Former weights
QScaler Initialization	Random
Learning Rate	$1\times 10^{-5}$
Optimizer	AdamW
Epochs	1
GPU Hardware	1 × NVIDIA RTX 4090 (24 GB)

**Table 2:** Cross-domain retrieval performance, memory usage, and throughput. COCO2014 is the in-domain dataset; MedPix and RSICAP are used for zero-shot evaluation. Throughput is computed as the reciprocal of average processing time per sample. Bold values indicate the best results for the corresponding metric.

Model	COCO	MedPix	RSICAP	Memory (MB)	Throughput (Images/s)
CLIP	0.2572	0.2531	0.2726	2880	250.00
DynamicViT	0.7982	-	0.5501	14,561	29.41
CrossGET	0.9387	0.3478	0.4981	16,648	33.33
BLIP2	0.8569	0.3473	0.5394	7213	52.63
Ours	**0.9411**	0.3231	0.4946	15,149	29.41

**Table 3:** Ablation of *QScaler* on *BLIP2-IDQ-Former*. Sim denotes average cosine similarity, R@5 denotes recall at rank 5, and MRR denotes mean reciprocal rank. The table also reports average query length, per-sample latency, and efficiency (FLOPs and throughput). FLOPs are computed on the *encode_image* path (vision encoder and Q-Former), and throughput is measured with batch size 1 under fp16. Arrows indicate the optimal direction of the metric (↑: higher is better; ↓: lower is better). Bold values indicate the best results for the corresponding metric.

Setting	Sim↑	R@5↑	MRR↑	Q-Len	FLOPs↓ (G)	Thr↑ (img/s)
With QScaler	**0.94**	0.34	0.25	17.80	**258.29**	**21.85**
Without QScaler	0.93	**0.35**	**0.26**	32.00	259.63	21.66

**Table 4:** Ablation of Q-Former training. Sim denotes average cosine similarity, R@5 denotes recall at rank 5, and MRR denotes mean reciprocal rank. The table also reports average query length, per-sample latency, and efficiency (FLOPs and throughput). FLOPs are computed on the *encode_image* path (vision encoder and Q-Former), and throughput is measured with batch size 1 in fp16 under the empirical distribution of query lengths. Arrows indicate the optimal direction of the metric (↑: higher is better; ↓: lower is better). Bold values indicate the best results for the corresponding metric.

Setting	Sim↑	R@5↑	MRR↑	QLen	FLOPs↓ (G)	Thr↑ (img/s)
Trainable Q-Former	**0.94**	**0.34**	**0.25**	17.80	258.29	21.85
Frozen Q-Former	0.93	0.27	0.20	16.42	**258.10**	**21.89**

## Data Availability

The original data presented in this study are openly available. COCO2014 is available at https://cocodataset.org/#home (accessed on 15 August 2025), MedPix is available at https://medpix.nlm.nih.gov/home (accessed on 15 August 2025), and RSICAP is available at https://github.com/Lavender105/RSGPT (accessed on 15 August 2025).
